# A Short-Term Biological Indicator for Long-Term Kidney Damage after Radionuclide Therapy in Mice

**DOI:** 10.3390/ph10020057

**Published:** 2017-06-21

**Authors:** Giovanni Pellegrini, Klaudia Siwowska, Stephanie Haller, Daniel J. Antoine, Roger Schibli, Anja Kipar, Cristina Müller

**Affiliations:** 1 Laboratory for Animal Model Pathology (LAMP), Institute of Veterinary Pathology, Vetsuisse Faculty, University of Zurich, 8057 Zurich, Switzerland; giovanni.pellegrini@uzh.ch (G.P.); anja.kipar@uzh.ch (A.K.); 2 Center for Radiopharmaceutical Sciences ETH-PSI-USZ, Paul Scherrer Institut, 5232 Villigen-PSI, Switzerland; klaudia.siwowska@psi.ch (K.S.); steffihaller@gmx.ch (S.H.); roger.schibli@psi.ch (R.S.); 3 Department of Molecular & Clinical Pharmacology, University of Liverpool, Liverpool L69 3GE, UK; d.antoine@liverpool.ac.uk; 4 Department of Chemistry and Applied Biosciences, ETH Zurich, 8093 Zurich, Switzerland

**Keywords:** radiation nephropathy, kidney, ^177^Lu, radiofolate, γ-H2AX, DNA double-strand breaks

## Abstract

Folate receptor (FR)-targeted radionuclide therapy using folate radioconjugates is of interest due to the expression of the FR in a variety of tumor types. The high renal accumulation of radiofolates presents, however, a risk of radionephropathy. A potential option to address this challenge would be to use radioprotectants, such as amifostine. Methods for early detection of kidney damage that—in this case—cannot be predicted based on dose estimations, would facilitate the development of novel therapies. The aim of this study was, therefore, to assess potentially changing levels of plasma and urine biomarkers and to determine DNA damage at an early stage after radiofolate application. The identification of an early indicator for renal damage in mice would be useful since histological changes become apparent only several months after treatment. Mice were injected with different quantities of ^177^Lu-folate (10 MBq, 20 MBq and 30 MBq), resulting in mean absorbed kidney doses of ~23 Gy, ~46 Gy and ~69 Gy, respectively, followed by euthanasia two weeks (>85% of the mean renal radiation dose absorbed) or three months later. Whereas all investigated biomarkers remained unchanged, the number of γ-H2AX-positive nuclei in the renal cortex showed an evident dose-dependent increase as compared to control values two weeks after treatment. Comparison with the extent of kidney injury determined by histological changes five to eight months after administration of the same ^177^Lu-folate activities suggested that the quantitative assessment of double-strand breaks can be used as a biological indicator for long-term radiation effects in the kidneys. This method may, thus, enable faster assessment of radiopharmaceuticals and protective measures by preventing logistically challenging long-term investigations to detect kidney damage.

## 1. Introduction

The concept of targeted radionuclide therapy takes an advantage of the expression of a tumor-associated receptor or antigen and a ligand for targeting that can be labeled with a therapeutic radionuclide. Radionuclide therapy using peptide-based and other small molecular-weight radioligands is already established in the clinics, however, only for a limited number of tumor types [1]. One of the challenges during the development of novel radionuclide therapies is the undesired accumulation of radioactivity in healthy organs and tissues. Among the organs at risk are the kidneys due to renal excretion of small molecular-weight radiopharmaceuticals [2].

The toxic effects of radiation to the kidneys are associated with a plethora of morphological and functional changes which are referred to as radiation nephropathy [3,4]. This is a chronic, progressive, irreversible condition that occurs in both humans and laboratory animals and is characterized by a late, but sudden onset of glomerular and tubular alterations eventually leading to end-stage kidneys [4]. Radiation nephropathy is associated with injury to all renal compartments, such as the glomeruli (glomerulosclerosis and mesangial proliferation), the tubulo-interstitial compartment (tubular atrophy and interstitial fibrosis) and the renal vasculature (occlusive thrombotic and non-thrombotic lesions) [4,5]. 

Over the years, different strategies were reported to overcome the problem of radiation-induced kidney toxicity, including application of diuretic agents to increase renal excretion of the compound or positively charged amino acids to decrease the accumulation of the radioactivity in kidneys [6,7]. An alternative option to reduce the risk of radionephropathy could be the application of radioprotective agents such as amifostine. It is applied as a prodrug and converted into an active radical scavenger by an alkaline phosphatase present in renal tissue [8]. The radioprotective mechanism of amifostine was also reported to be associated with rapid oxygen consumption, resulting in cellular anoxia, and thus, reduced effects of radiation [8]. Rolleman et al. have shown that the treatment of rats with radiolabeled somatostatin analogs in combination with amifostine significantly decreased kidney damage in comparison with radionuclide therapy alone [9]. Amifostine-treated animals showed absence of body weight loss, lower histopathological scores of kidney damage and higher uptake of ^99m^Tc-DMSA in the kidney indicative of intact renal function [9]. Amifostine (Ethyol^TM^) is the only radioprotectant approved for clinical use so far; however, investigations of other radioprotective agents, including blockers of oxygen consumption, free-radical scavengers, DNA repair boosters and others are ongoing [10].

The folate receptor alpha (FR) is expressed in many tumor types of epithelial origin, most prominently in ovarian, endometrial and lung cancer [11,12]. The development of FR-targeted radionuclide therapy using folic acid conjugates of therapeutic nuclides would, therefore, be of high interest. The potential of this therapy concept has been demonstrated in preclinical settings [13]; however, translation to the clinics was not feasible so far, due to the high renal accumulation of radiofolates, which poses the risk of damage to the kidneys. Several attempts to reduce the kidney uptake of radiofolates were made in past years, among those also the use of agents which may modulate the tissue distribution [14]. The concept of using kidney-selective radioprotective agents has not been investigated so far in combination with radiofolates even though it seems promising. 

Histological features consistent with radiation nephropathy in laboratory animals are observed only months after external beam radiation or radionuclide therapy, while the early stages after radiation exposure are dominated by subtle histological changes that vary between different species [4,15]. In general, the majority of markers of radiation-induced kidney damage are visible only weeks or even months after start of the therapy [4]. Assessment of murine radiation nephropathy requires, therefore, long-term in vivo experiments, which are logistically challenging.

It is commonly accepted that an absorbed kidney dose of 23 Gy is the safe upper limit [16]. The physically calculated dose does, however, not take the action of radioprotective agents into account, hence, the dose calculation in such case is not a reliable measure to predict long-term effects after radionuclide therapy. The establishment of methods that allow determination of radiation effects in the renal tissue predictive for long-term damage would enable more rapid development of protective measures such as the use of antioxidants. 

In this study, renal tissue sections of mice that were euthanized two weeks or three months after injection of different activities of ^177^Lu-folate were investigated histologically and an immunohistochemical analysis of phosphorylated histone H2AX (γ-H2AX), a double-strand break (DSB) marker [17], was performed. Quantification of γ-H2AX may possibly allow determination of the degree of kidney irradiation which would cause long-term renal damage and serve as a valid marker for the protective effect of radical scavengers and other radioprotective agents. In this study, we also analyzed urine and plasma samples of the same mice. These included the determination of commonly used endogenous markers of renal function, such as plasma levels of blood urea nitrogen and creatinine, as well as urinary biomarkers considered to indicate acute tubular injury.

## 2. Results and Discussion

One of the main advantages of radionuclide therapy is the possibility to deliver a radioactive source to the malignant tissue by targeting a tumor-specific receptor or antigen [1]. Organs other than the target tissues, however, may be exposed to radiation as well, as occurs in the kidneys due to renal excretion of the radioconjugates [7,18,19,20]. Accordingly, undesired side effects of radionuclide therapy often include chronic, slowly progressing loss of kidney function [2]. This is particularly true in the case of folate-based radioconjugates as they bind specifically to the FR, expressed in the renal proximal tubules [21]. The development of an early marker that could be predictive of late renal radiation-induced injury would be of major benefit for the assessment of radiopharmaceutical candidates in preclinical studies. In the present study, we investigated DSBs as short-term indicators of radiation effects which may serve as a measure for kidney damage when dose calculations cannot be applied. In addition, we determined the morphological changes in the renal tissue (Table 1) and assessed urine and blood plasma parameters, elevated in acute kidney injury (Supplementary Materials, Table S1), of mice two weeks and three months after application of ^177^Lu-folate.

### 2.1. Effect of ^177^Lu-Folate Treatment on Body Weights of Mice

**Figure 1:**
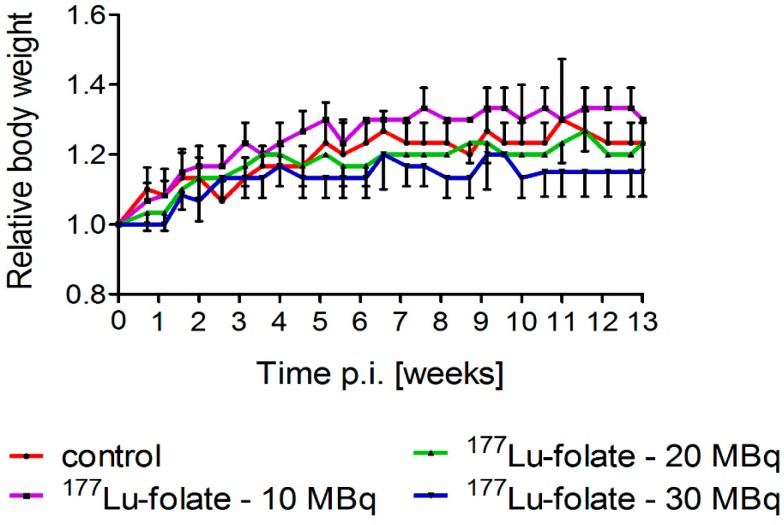
Relative body weights of animals treated with different activities of ^177^Lu-folate (10 MBq, 20 MBq or 30 MBq). Data points represent an average of six mice per group up to two weeks and of three mice per group from Week 2 until Week 13 post treatment.

Groups of six mice received either only saline or ^177^Lu-folate at different activities (10 MBq, 20 MBq and 30 MBq), corresponding to mean absorbed kidney doses of 23 Gy, 46 Gy and 69 Gy, respectively (Table 2) [22]. The body weight and general health status of mice were monitored over the whole study period. Directly before treatment of the mice, the average body weights (19.8–20.4 g) did not differ significantly between the various groups of mice. The weight gain over the first two weeks was similar in all groups (0.7–17%) resulting in RBWs of 1.17 ± 0.06, 1.13 ± 0.06 and 1.07 ± 0.06 in mice that received 10 MBq, 20 MBq and 30 MBq ^177^Lu-folate, respectively, and 1.13 ± 0.06 in untreated control mice. After three months, the RBW of mice increased to 1.15–1.30 (Figure 1). These results indicated that ^177^Lu-folate did not have any effect on the body weight of the mice in the first three months, which is in accordance with long-term studies, where the body weight of control mice and treated mice remained stable over the whole study period of eight months [15]. Only the mice treated with high activity of ^177^Lu-folate (30 MBq) were found to progressively lose body weight from week 15 onwards and reached endpoint criteria which required euthanasia between week 15 and 26 post treatment [15].

### 2.2. Effect of ^177^Lu-Folate Treatment on the Levels of Urine and Plasma Biomarkers

Urinary markers, such as kidney injury molecule-1 (Kim-1), neutrophil gelatinase-associated lipocalin (NGAL), *N*-acetyl-β-d-glucosaminidase (NAG) and interleukin-18 (IL-18) have been previously reported to rise as a result of acute tubular injury [23,24]. In our study, the levels of these biomarkers remained, however, unchanged in all groups of mice independent of the applied dose. Cystatin C was the only parameter that increased slightly in treated mice that received high activity of ^177^Lu-folate (30 MBq) as compared to control mice and mice which received lower activities (131–145 ng/mg CRE) (Supplementary Materials, Table S1). The urinary levels of cystatin C were reported to rise after tubular injury [25,26,27]. Increased levels of cystatin C, in the absence of changes of other markers of acute kidney injury, have been previously described also in rats, where a 3-fold increase in cystatin C compared to the control was detected 24 h after exposure to total body irradiation with x-rays [28]. However, in our study, the cystatin C levels did not change significantly and, even though the cohort of mice was very small, it is unlikely that cystatin C could be used as a biological indicator for the prediction of long-term radionephropathy.

Investigation of plasma samples from mice euthanized two weeks after injection of ^177^Lu-folate also did not show significant changes in blood urea nitrogen (BUN), creatinine (CRE) and Kim-1 levels. These results indicate that the plasma biomarkers used in this study cannot be employed for prognostication of radiation-induced long-term renal damage.

### 2.3. Renal Weight and Histopathological Observations

In a previous study, in which the long-term damage of kidneys was investigated, a significant reduction in renal size was reported at five to eight months after treatment with ^177^Lu-folate [22]. A kidney dose of 23 Gy resulted in an absolute kidney weight reduction of 23% as compared to control mice after eight months. Higher mean absorbed kidney doses (46 Gy and 69 Gy) resulted in renal weight decreases of 32% and 61%, respectively (unpublished data). These findings are in agreement with those of clinical studies, where a progressive decrease in kidney size was observed over a period of eight years in patients that had undergone abdominal radiation therapy [29]. 

In mice euthanized two weeks after administration of 20 MBq and 30 MBq ^177^Lu-folate, a slight, but not significant reduction of kidney weight was noted as compared to the kidney weight of control mice and mice injected with 10 MBq ^177^Lu-folate. In mice that were euthanized after three months, there was no weight difference in the kidneys, compared to those of the concurrent controls. This indicates that renal weights at these early time points are not indicative of long-term renal injury.

**Figure 2:**
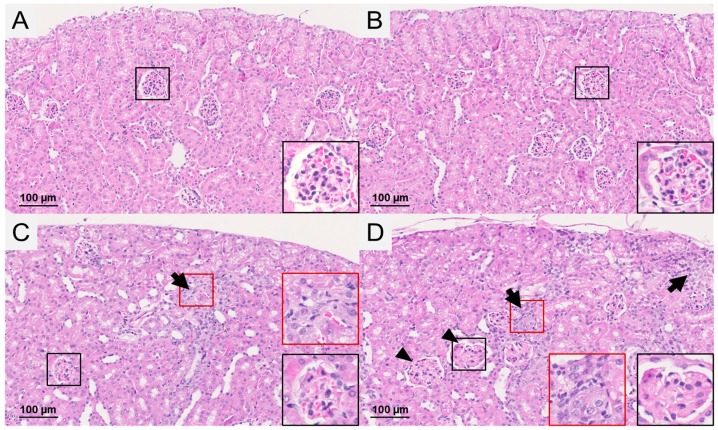
Histological findings in the renal cortex of mice euthanized three months after injection of (**A**) saline, (**B**) 10 MBq ^177^Lu-folate, (**C**) 20 MBq ^177^Lu-folate and (**D**) 30 MBq ^177^Lu-folate. (**A**/**B**) No histological changes were observed in the control and low-dose group. (**C**) In the mid-dose group, changes were restricted to the presence of scattered groups of degenerated tubules (arrow) in one of the three examined animals (final score 1). (**D**) In the high-dose group, mild kidney injury was observed (3/3 mice, final score 2) represented by multifocal tubular degeneration (arrows) and slight enlargement of glomeruli with fewer capillaries (arrowheads). Higher magnification of representative glomeruli (black squares) and tubular lesions (red squares) indicated by arrows (insets are 5× magnified as compared to the images).

At two weeks after application of ^177^Lu-folate, kidneys did not exhibit any histological changes (Table 1, Supplementary Materials, Figure S1). This was also true for the kidneys of mice euthanized three months after application of ^177^Lu-folate at an activity of 10 MBq, resulting in a mean absorbed kidney dose of 23 Gy (final score 0, determined according to the scoring system shown in Table 3). In mice which received 20 MBq ^177^Lu-folate, resulting in a mean absorbed kidney dose of 48 Gy, slight renal damage consisting of scattered groups of degenerated tubules was observed in one of three mice, resulting in a final score of 1. However, no damage was observed in the two other mice receiving the same activity of ^177^Lu-folate (final score 0) (Figure 2, Table 1). Mild nephropathy with a final score of 2 was detected in mice that had received high activity of ^177^Lu-folate (30 MBq) resulting in a mean absorbed kidney dose of 69 Gy (Figure 2, Table 1). This was characterized by deposition of proteinaceous material and a reduced number of capillaries in the glomeruli, as well as multifocal cortical tubular degeneration and collapse. These results are in line with those of the previously performed long-term study [15], where mice that received high activities of ^177^Lu-folate (30 MBq) had to be euthanized five or six months after treatment due to progressive body weight loss and signs of unease. Morbidity was attributed to radiation nephropathy with an average score 4 (Table 1). 

The histological data indicate that the earliest evidence of radiation-induced renal injury after injection of ^177^Lu-folate can be observed after three months, as low grade (minimal or mild) renal damage in the high-dose group and, to a minimal extent, the mid-dose group. These findings are in agreement with the onset of a dose-dependent renal damage around this time after ^177^Lu-folate treatment, which obviously progresses with time, leading to the degree of damage seen at later stages [15].

### 2.4. Immunohistological Detection of DNA Double-Strand Breaks

DNA damage is the first manifestation of radiation effects in tissue [30]. Among the proteins involved in the early steps of the cellular response to DNA damage is γ-H2AX, the phosphorylated form of the histone protein H2AX. Over many years γ-H2AX has been used as a marker to monitor the formation and persistence of DNA DSBs and the reparative response to DNA damage for the evaluation of the efficacy of cancer therapies, as well as a tool to address radiobiological questions and investigations using biodosimetry [17,31,32,33,34]. It has previously been reported that γ-H2AX is detectable very early after in vitro exposure of cells to ionizing radiation, reaching maximum levels within 15–30 min after irradiation, followed by a decline over several hours due to rejoining of the DSBs [35]. A decline of γ-H2AX positive cells in tissues, including the kidneys of mice irradiated with x-rays, has been reported to occur within ~23 h [36]. Nevertheless, γ-H2AX levels were analyzed in this study after absorption of >85% of the radiation dose which, according to the biodistribution data previously obtained with the ^177^Lu-folate, is reached about 14 days after radiofolate injection [22].

**Figure 3:**
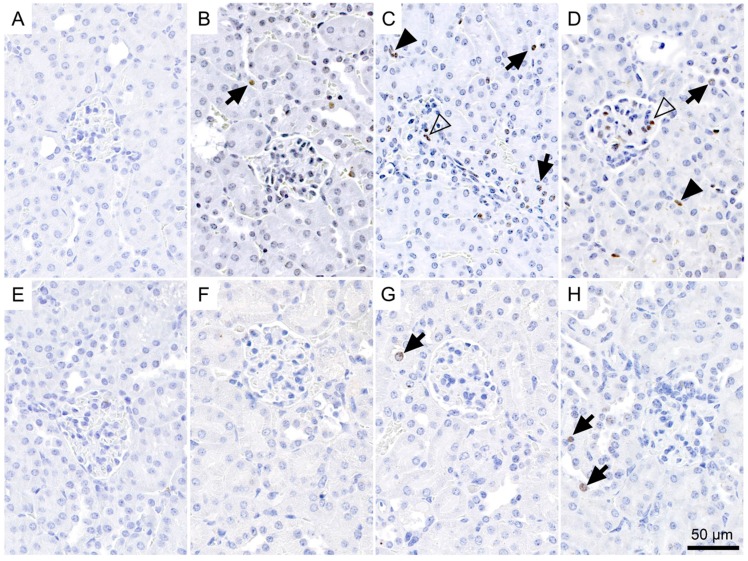
Incidence of γ-H2AX-positive nuclei in the renal cortex of mice euthanized two weeks after the injection of (**A**) saline, (**B**) 10 MBq, (**C**) 20 MBq and (**D**) 30 MBq ^177^Lu-folate, or three months after injection of (**E**) saline, (**F**) 10 MBq, (**G**) 20 MBq, and (**H**) 30 MBq ^177^Lu-folate. Positive nuclei typically exhibited one to multiple intensely brown, round foci of approximately 1 µm in diameter, or a more diffuse light brown staining. The number of γ-H2AX-positive nuclei is markedly increased in a dose-related manner in mice at two weeks (**B**–**D**), and to a lesser extent and only in the mid- and high-dose groups in mice at three months (**G**,**H**). γ-H2AX-positive nuclei in tubular epithelial cells, glomeruli and interstitium are indicated with arrows, open arrowheads and solid arrowheads, respectively.

**Figure 4:**
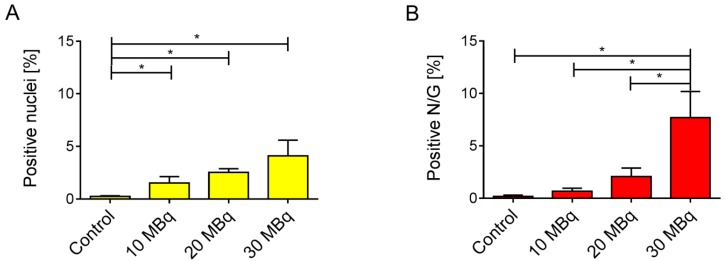
(**A**) Percentage of γ-H2AX-positive nuclei and (**B**) percentage of positive nuclei per glomerulus (N/G) in kidneys of mice two weeks after injection of ^177^Lu-folate. Compared to the kidneys of untreated mice which showed rare positive cells, there is a dose-related, statistically significant increase in the number of γ-H2AX-positive nuclei in mice euthanized two weeks after injection (≥10 MBq ^177^Lu-folate). The highest activity of ^177^Lu-folate was associated with a statistically significant increase in the number of γ-H2AX-positive nuclei in glomerular cells at the two-week time point (B). *p < 0.05.

We determined a significant increase in the proportion of γ-H2AX-positive nuclei in the renal cortex of all treated groups as compared to the low basal level seen in the untreated controls two weeks after injection of ^177^Lu-folate (Figure 3A–D and Figure 4A). The presence of γ-H2AX was generally rare in cells within the glomerulus. An exception were the kidneys of mice that received high activities of ^177^Lu-folate (30 MBq), in which the positive nuclei count limited to the glomerular area revealed a prominent, statistically significant increase of γ-H2AX (Figure 3).

In the renal cortices of mice euthanized two weeks after injection of 10 MBq, 20 MBq and 30 MBq ^177^Lu-folate, ~2.7, ~4.1 and ~7.5 (~1.5%, ~2.5% and ~4.1% of the total nuclei) γ-H2AX**-**positive nuclei per high-power field were counted, respectively (Figure 4). In contrast, the kidneys of untreated mice exhibited very low levels of γ-H2AX-positive nuclei (~0.1 nuclei per high-power field corresponding to ~0.2% of total nuclei). The increase in the number and proportion of γ-H2AX-positive nuclei was significant and clearly dose-related. These findings were in agreement with dose-dependent radiation effects in tissue caused by external radiation therapy [37]. Importantly, dose-dependent increase in the number of DSBs was seen in the absence of microscopic changes in the kidneys, that is, long before morphological evidence of radiation nephropathy (Supplementary Materials, Figure S1). 

A clearly less obvious result was obtained in mice euthanized three months after injection of ^177^Lu-folate (Figure 3E–H), as the amount of DSBs in kidney tissues of treated mice was comparable to the basal level. 

The strong, dose-dependent γ-H2AX formation in the kidneys two weeks after radiofolate injection indicated that γ-H2AX represents a potentially useful marker for the early evaluation of radiation nephropathy. The time point for determination of DSB cannot be seen as a best after two weeks, but would critically depend on the tissue distribution of a particular radioligand and the residence time in the kidneys.

Glomeruli of mice exposed to low activities of ^177^Lu-folate showed negligible nuclear γ-H2AX staining similar to control mice. The highest activity of ^177^Lu-folate (30 MBq, resulting in a mean absorbed kidney dose of 69 Gy) was, however, associated with a sharp, statistically significant increase in the formation of γ-H2AX foci in glomerular cells at the two-week time point. In a previous long-term study [15], severe widespread glomerulosclerosis was observed in mice that had received the same activity, euthanized five to six months after ^177^Lu-folate application, resulting in an average partial glomerular score of 5 [15]. This was more severe than the kidney damage in mice euthanized eight months after treatment with 10 MBq and 20 MBq ^177^Lu-folate, where the average histological glomerular injury score was 2.0 and 3.8, respectively. 

### 2.5. Correlation of Biological Markers with a Long-Term Kidney Damage

**Figure 5:**
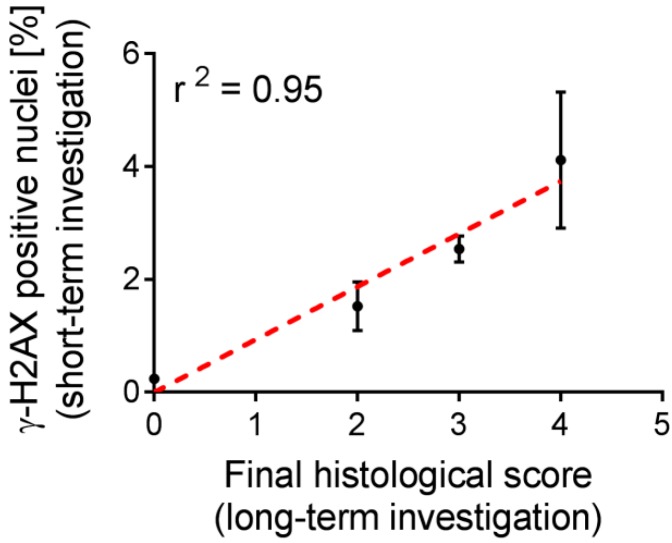
Positive correlation between the number of γ-H2AX-positive nuclei in the kidneys of mice treated with different activities of ^177^Lu-folate (10 MBq, 20 MBq and 30 MBq) and untreated control mice after two weeks and long-term final scores of histopathological changes. Dose-related increases in the number of γ-H2AX-positive nuclei, as a biological indicator of radiation effects, correlated well with long-term kidney damage.

It is known from the literature, that protective agents may influence the effect of radiation on the tissue. As a result, the calculated dose of applied activity may not be an accurate measure to predict the toxicity of such therapy, thus, biological markers would be of high relevance [9]. The functional and morphological aspects of chronic nephrotoxicity, induced by the treatment with ^177^Lu-folate in mice, were assessed in a previous study [15,22] and used herein to assess the value of short- and mid-term biological indicators of radiation effects as investigated in the present study. In the studies by Haller et al. [15,22], nude mice were monitored over a period of five to eight months after the application of various activities of ^177^Lu-folate (10 MBq, 20 MBq and 30 MBq per mouse). Striking dose-dependent nephrotoxicity was indicated by decreased renal uptake of ^99m^Tc-DMSA over time, increased levels of BUN and CRE as well as marked cortical scarring and glomerulosclerosis determined in the kidneys of mice after euthanasia. The quantification of γ-H2AX, investigated two weeks after injection of ^177^Lu-folate, correlated strongly with the long-term kidney damage (r^2^ = 0.95), represented by the final histological score previously determined by Haller et al. (Figure 5) [15].

In the context of the present study, caution is warranted when trying to identify cutoff levels of γ-H2AX formation predictive for long-term radionephropathy as the sample size was much too small to yield quantitatively reliable results. However, in terms of directions for future research, it will be important to identify robust threshold levels of γ-H2AX, as a biological indicator of radiation effects independent of the calculated dose, for a rapid evaluation of kidney toxicity in studies where radionuclide therapy will be combined with radioprotectants.

## 3. Conclusions

Increased numbers of γ-H2AX nuclei, determined two weeks after application of ^177^Lu-folate when >85% of the mean radiation dose to the kidneys was absorbed, correlated well with the severity of long-term renal damage. 

Our results suggest that quantification of nuclear phosphorylated H2AX as a biological indicator for radiation effects independent of the calculated absorbed dose may be useful to predict long-term renal damage and could facilitate the development of methods using radioprotectants in combination with targeted radionuclide therapies. The proposed concept is likely translatable from radiofolates to the application of other radiopharmaceuticals where kidneys are at risk to be damaged. Further and more detailed preclinical studies are warranted to corroborate this outcome and investigate the concept in more detail.

## 4. Materials and Methods 

### 4.1. Preparation of ^177^Lu-folate

The DOTA-folate conjugate containing an albumin-binding entity (cm09, herein referred to as “folate” [21]) was previously developed in our group. No-carrier added ^177^LuCl_3_ was kindly provided by Isotope Technologies Garching (ITG GmbH, Garching, Germany). Radiolabeling of the folate conjugate was performed in a mixture of HCl (0.05 M) and Na-acetate (0.5 M) at pH 4.5 and elevated temperature (10 min at 95 °C). Quality control of ^177^Lu-folate was carried out using high-performance liquid chromatography (HPLC) as previously reported [21]. The radiolabeling of the folate compound at the specific activity of up to 20 MBq/nmol was achieved with a radiochemical purity of >97%.

### 4.2. Animal Experiments

Animal experiments were approved by the local veterinary authorities and conducted in accordance with the Swiss legislation for animal experiments. Female athymic nude mice (Crl:CD1-Foxn1^nu^, 5–7-week-old) were purchased from Charles River Laboratories (Sulzfeld, Germany). Animals were fed with a folate-deficient rodent diet (ssniff Spezialdiäten GmbH, Soest, Germany), starting one week prior to the injection of the radiofolate. Mice were divided into four groups of six animals each and were intravenously (i.v.) injected with saline or ^177^Lu-folate at therapeutic quantities of activity (10 MBq, 20 MBq and 30 MBq, respectively). The determination of the mean absorbed dose to the kidneys has been reported previously (Table 2) [22]. 

End-point criteria were defined as weight loss of >15% of the initial body weight and/or signs indicating pain and unease. Body weights were measured twice a week for the determination of the relative body weight (RBW = W_X_/W_0_; with W_X_ = body weight at day x and W_0_ = body weight at day 0) and indicated as average RBW for each group. Animals of each group (n = 6) were euthanized two weeks (n = 3) or three months (91 days, n = 3) after injection of the ^177^Lu-folate, respectively. The long-term effects to the kidneys after treatment of mice with ^177^Lu-folate were previously evaluated over a period of 5–8 months and reported by Haller et al. [15,22]. Data from these experiments were used in the present study for comparative purposes.

### 4.3. Determination of Plasma and Urinary Markers

Analysis of plasma and urinary markers is described in Supplementary Materials. Investigated markers included creatinine and blood urea nitrogen, kidney injury molecule-1 (KIM-1, designed as Kim-1 in rodents), neutrophil gelatinase-associated lipocalin (NGAL), *N*-acetyl-β-d-glucosaminidase (NAG), cystatin C and interleukin-18 (IL-18) [38].

### 4.4. Necropsy, Tissue Processing and Histological Examination

All animals were euthanized by exsanguination after CO_2_ asphyxiation. After dissection of the animals and a full macroscopic examination, the kidneys were weighed and fixed in 4% neutral-buffered formalin (Formafix, Hittnau, Switzerland). Kidneys were trimmed (longitudinal and cross-sections of both kidneys), dehydrated through graded alcohols and routinely paraffin wax embedded. Consecutive sections (3–5 µm) were prepared, mounted on glass slides and routinely stained with hematoxylin eosin (HE) or subjected to immunohistochemical staining. Renal injury was assessed and scored in HE-stained sections, as previously reported (Table 3) [15]. 

### 4.5. Immunohistochemistry for the Detection of DNA Double-Strand Breaks

The immunohistochemical staining for the phosphorylated γ-H2AX histone antigen served for the determination of DNA double-strand breaks (DSBs) [39]. Briefly, sections were deparaffinized in xylene and rehydrated through graded ethanol. For antigen retrieval, sections were incubated in 10 mM Tris-EDTA buffer (pH 9.0) for 15 min at 98 °C. This was followed by incubation with rabbit anti-mouse γ-H2AX antibody (polyclonal antibody #2577, Cell Signaling Technology, Danvers, MA, USA, 1:50 dilution in Dako antibody diluent, Dako-Agilent Technologies, Denmark) overnight at 4 °C. Afterwards, the slides were incubated for 30 min with a horseradish peroxidase (HRP)-labeled polymer, conjugated to a secondary anti-rabbit antibody (Dako Envision^TM^ System, Dako-Agilent Technologies). The reaction was visualized using 3,3’-diaminobenzidine (DAB) as chromogen, followed by light counterstain with hematoxylin. The immunohistochemical staining was performed using an Autostainer (Dako Autostainer Universal Staining System Model LV-1, Dako-Agilent Technologies). A formalin-fixed, paraffin-embedded human tumor xenograft obtained after irradiation of a tumor-bearing mouse served as positive control, and kidney sections incubated with the dilution buffer without primary antibody served as negative controls. 

All labeled slides were scanned using a digital slide scanner (NanoZoomer-XR C12000; Hamamatsu, Japan), and the number of nuclei exhibiting staining for γ-H2AX was calculated in the digital slides using the Visiopharm Integrator System (VIS, version 4.5.1.324, Visiopharm, Hørsholm, Denmark). Briefly, 30 regions of interest (ROIs) with a size of 0.237 mm^2^ (the area of a high-power field with one ocular of 22 mm field of view), were randomly selected across the renal cortex of all animals. A threshold classification allowed recognition of positive (brown) and negative nuclei in each ROI, and the results were expressed as % of positive nuclei per ROI. To selectively measure the amount of positive nuclei in the glomeruli, the same analysis was carried out on ROIs that were created by manual drawing over 30 consecutive glomeruli per kidney. The quantitative analysis was performed by two independent investigators and expressed as the average of the two sets of data. The automated system was validated based on the numbers obtained in the same ROI by manual counting by an investigator.

### 4.6. Statistical Analysis

Data are presented as mean ± standard. Statistical analyses were conducted using one-way ANOVA with Bonferroni’s multiple comparison post-test (GraphPad Prism, version 5.01).

## Figures and Tables

**Table 1:** Histological evaluation of ^177^Lu-folate induced renal changes in mice.

^177^Lu-folate (MBq)	Time of Euthanasia	Score Glomeruli (Average)	Score Tubules (Average)	Score Interstitium (Average)	Cumulative Score	Final Score *
Results of the present study						
-	2 weeks	0	0	0	0	0
10		0	0	0	0	0
20		0	0	0	0	0
30		0	0	0	0	0
-	3 months	0	0	0	0	0
10		0	0	0	0	0
20		0	0.3	0	0.3	0 (n = 2); 1 (n = 1)
30		2.0	1.5	0	3.5	2
Results of a previous study reported by Haller et al. [15]						
-	8 months	0	0	0	0	0
10		2.0	1.5	0.4	3.9	2
20		3.8	3.3	3.3	10.4	3
30	5–6 months **	5	4.2	4.6	13.8	4

* In the HE-stained sections, each renal compartment was evaluated separately and given a partial score ranging from 0 to 5. Partial scores were then summed (=cumulative score) and converted to a final score, indicating the degree of renal injury according to Table 3 [15]. ** Mice reached an endpoint criterion (body weight loss) between 5 and 6 months after ^177^Lu-folate application.

**Table 2:** Mean absorbed kidney doses in nude mice after the injection of different quantities of ^177^Lu-folate calculated in previous studies [22].

	Saline	^177^Lu-folate		
Injected activity (MBq)	-	10	20	30
Kidney dose (Gy)	-	~23	~46	~69

**Table 3:** Scoring scheme for the assessment of renal injury [15]. A cumulative score, obtained by the addition of scores for the changes in each compartment (glomeruli, tubules and interstitium) was converted to a final score as an indicator of the overall degree of renal injury.

Cumulative Score (Glomeruli, Tubules, Interstitium)	Final Score	Renal Injury
0–0.9	0	no histological abnormality
1.0–2.9	1	minimal
3.0–6.9	2	mild
7.0–10.9	3	moderate
11.0–13.9	4	marked
14.0–15.0	5	severe

## Supplementary Materials

The following are available online at http://www.mdpi.com/1424-8247/10/2/57/s1, Table S1: Urine biomarkers of acute renal injury in ^177^Lu-folate treated mice, Figure S1: Histological findings in HE-stained kidney tissue sections of mice euthanized two weeks after injection of saline, 10 MBq ^177^Lu-folate, 20 MBq ^177^Lu-folate and 30 MBq ^177^Lu-folate.
