# Supplementary material for: A Short-Term Biological Indicator for Long-Term Kidney Damage after Radionuclide Therapy in Mice
Source: Pharmaceuticals (Basel). 2017 Jun 21;10(2):57. doi: 10.3390/ph10020057 (PMC5490414; doi:10.3390/ph10020057)
Supplement: Supplementary file 1 [file pharmaceuticals-10-00057-s001.pdf]

pharmaceuticals

Supplementary Materials

A Short-Term Biological Indicator for Long-Term Kidney Damage after Radionuclide Therapy in Mice

Giovanni Pellegrini, Klaudia Siwowska, Stephanie Haller, Daniel J Antoine, Roger Schibli, Anja Kipar, and Cristina Müller

1. Determination of Urine and Plasma Biomarkers

1.1. Experimental

After provoking mice to urinate by applying gentle, abdominal pressure over the urinary bladder, the urine was collected using a pipette. Subsequently, blood was sampled from the retrobulbar vein under isoflurane inhalation anesthesia prior to euthanasia of the mice. Blood samples were centrifuged (4 °C, 20 min, 1600 rpm), and the plasma was collected. Both urine and plasma samples were stored at −20 °C until analysis.

After thawing, plasma and urine samples were centrifuged (3,000 rpm, 5 min) and the supernatants were used for biomarker measurements as previously reported or according to manufacturer's assay instructions [1,2]. The panel included the determination of blood urea nitrogen (BUN), creatinine (CRE) and kidney injury molecule-1 (Kim-1) in the plasma as well as Kim-1, neutrophil gelatinase-associated lipocalin (NGAL), *N*-acetyl-β-D-glucosaminidase (NAG), cystatin C, IL-18 and albumin in the urine samples. The results for urinary biomarkers were standardized for urinary creatinine and presented as the amount of biomarker per milligram of creatinine (mgCRE). Results for blood plasma biomarkers were presented as the amount of biomarker per volume.

Data are presented as mean ± standard. Statistical analyses were conducted using one-way ANOVA with Bonferroni's multiple comparison post-test (GraphPad Prism, version 5.01).

1.2. Results and Conclusion

Mice euthanized at the short-term endpoint of two weeks showed no changes in urinary cystatin C levels (131–145 ng/mgCRE) after treatment with low and medium activities of ^{177}Lu-folate (10 MBq and 20 MBq, respectively); however, an increased value (181 ng/mgCRE) was observed in mice treated with high activity of ^{177}Lu-folate (30 MBq) (Table S1). Increased levels of cystatin C, in the absence of changes of other markers of acute kidney injury, have been previously described also in rats 24 h after exposure to total body irradiation with x-rays [3], suggesting that cystatin C represents a sensitive marker of radiation-induced nephrotoxic damage. Indeed, after tubular injury, the urinary levels of cystatin C are known to be increased [4–6]. It is, thus, likely that the increased cystatin C level indicates tubular injury, as cystatin C is filtered by the glomerulus and metabolized by the proximal tubule cells. The other markers, such as Kim-1, NGAL, NAG and IL-18 have been previously reported to rise as a result of acute tubular injury, when analyzed within hours or days after therapy, rather than after two weeks as in the present study [7,8]. In our study, the levels of Kim-1, NGAL, NAG and IL-18 remained, however, unchanged in all groups of mice.

Table S1. Urine biomarkers of acute renal injury in mice treated with 10 MBq, 20 MBq and 30 MBq ^{177}Lu-folate, respectively, and control mice which received only saline.

**^{177}Lu-folate [MBq]**	**Endpoint**	**Biomarkers levels in urine samples**				
		**Cystatin C (ng/mgCRE)**	**Kim-1 (ng/mgCRE)**	**NGAL (ng/mgCRE)**	**NAG (nu/mgCRE)**	**IL-18 (pg/mgCRE)**
-	2 weeks	140 ± 9.3	1 ± 0.5	126 ± 86.2	16.5 ± 5.8	254 ± 83.2
10		131 ± 12.5	1.6 ± 0.6	96.3 ± 18.8	16.2 ± 4.0	173 ± 19.9
20		145 ± 20.0	1.3 ± 0.6	188 ± 69.1	25.2 ± 1.9	217 ± 79.5
30		181 ± 10.2 *	1.2 ±0.4	105 ± 20.2	22.3 ± 10	250 ± 53.7
-	3 months	174 ± 6.6	1.7 ± 0.4	84.5 ± 34.5	16.7 ± 6.4	197 ± 30.9
10		135 ± 13.7	1.2 ± 0.3	167 ± 46.1	17.9 ± 6.6	200 ± 25.5
20		147 ± 16.2	1.4 ± 0.7	130 ± 74.5	18.6 ± 10.3	210 ± 68.1
30		185 ± 4.5	1.0 ± 0.4	140 ± 23.2	21.5 ± 8.1	199 ± 83.7

* significantly increased as compared to the control group ($p < 0.05$)

Investigation of plasma samples from mice euthanized two weeks after injection of ^{177}Lu-folate did not show significant changes in BUN levels in treated animals (29–43 mg/dL) as compared to untreated controls (38.2 ± 4.7 mg/dL). The same held true for CRE which was in the same range for treated animals (40–43 µmol/L) and untreated controls (42.8 ± 8.4 µmol/L) as well as for Kim-1 levels that showed values in the range of 135–143 pg/mL in treated animals versus 132 ± 20.8 pg/mL in controls. These results indicate that the plasma biomarkers used in this study cannot be employed for prognostication of radiation-induced long-term renal damage.

2. Histological Investigations

2.1. Experimental

Experimental procedure is described in the main manuscript.

2.2. Results and Conclusion

Results and conclusion are described in the main manuscript.

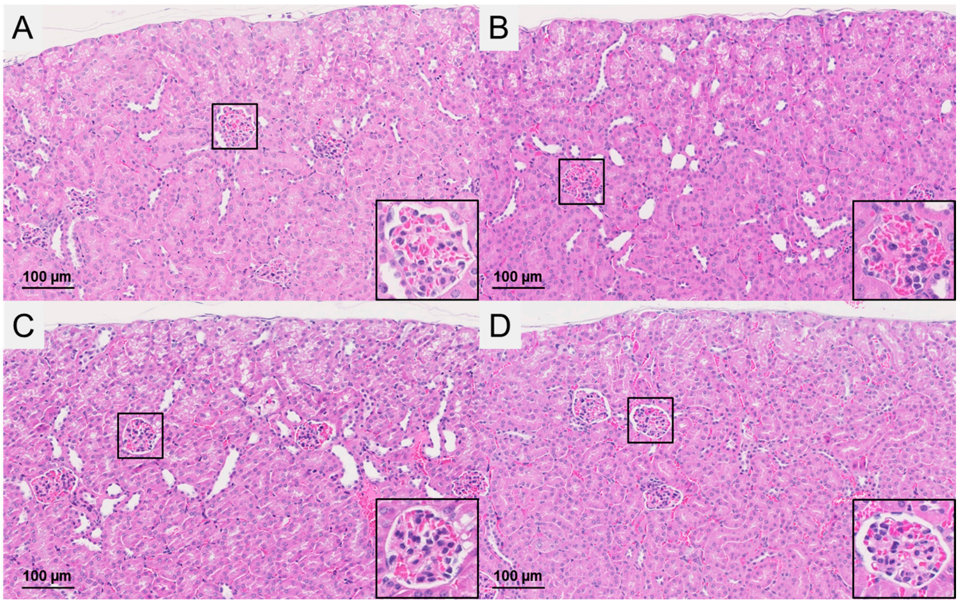

Figure S1. Histological findings in HE-stained tissue sections from the kidneys of mice euthanized two weeks after injection of (**A**) saline, (**B**) 10 MBq ^{177}Lu-folate, (**C**) 20 MBq ^{177}Lu-folate and (**D**) 30 MBq ^{177}Lu-folate. There was no evidence of renal injury regardless of treatment. All images represent HE-stained sections of renal cortices and include a high magnification of representative glomeruli (black squares) and adjacent tubules (insets are 5x magnified as compared to the images).

References

1. McWilliam, S.J.; Antoine, D.J.; Sabbisetti, V.; Turner, M.A.; Farragher, T.; Bonventre, J.V.; Park, B.K.; Smyth, R.L.; Pirmohamed, M. Mechanism-based urinary biomarkers to identify the potential for aminoglycoside-induced nephrotoxicity in premature neonates: A proof-of-concept study. *PloS ONE* **2012**, *7*, e43809.
2. Antoine, D.J.; Sabbisetti, V.S.; Francis, B.; Jorgensen, A.L.; Craig, D.G.; Simpson, K.J.; Bonventre, J.V.; Park, B.K.; Dear, J.W. Circulating kidney injury molecule 1 predicts prognosis and poor outcome in patients with acetaminophen-induced liver injury. *Hepatology* **2015**, *62*, 591-599.
3. Sharma, M.; Halligan, B.D.; Wakim, B.T.; Savin, V.J.; Cohen, E.P.; Moulder, J.E. The urine proteome as a biomarker of radiation injury. *Proteomics Clin. Appl.* **2008**, *2*, 1065-1086.
4. Herget-Rosenthal, S.; van Wijk, J.A.; Brocker-Preuss, M.; Bokenkamp, A. Increased urinary cystatin c reflects structural and functional renal tubular impairment independent of glomerular filtration rate. *Clin. Biochem.* **2007**, *40*, 946-951.
5. Koyner, J.L.; Bennett, M.R.; Worcester, E.M.; Ma, Q.; Raman, J.; Jeevanandam, V.; Kasza, K.E.; O'Connor, M.F.; Konczal, D.J.; Trevino, S., *et al.* Urinary cystatin c as an early biomarker of acute kidney injury following adult cardiothoracic surgery. *Kidney Int.* **2008**, *74*, 1059-1069.
6. Andreucci, M.; Faga, T.; Riccio, E.; Sabbatini, M.; Pisani, A.; Michael, A. The potential use of biomarkers in predicting contrast-induced acute kidney injury. *Int. J. Nephrol. Renovasc. Dis.* **2016**, *9*, 205-221.
7. Devarajan, P. Biomarkers for the early detection of acute kidney injury. *Curr. Opin. Pediatr.* **2011**, *23*, 194-200.
8. Edelstein, C.L. Biomarkers of acute kidney injury. *Adv. Chronic Kidney Dis.* **2008**, *15*, 222-234.
